# Compliance with medical regimen among hematological cancer patients and its association with symptoms of posttraumatic stress disorder and adjustment disorder

**DOI:** 10.3389/fpsyg.2023.1278485

**Published:** 2023-11-03

**Authors:** Franziska Springer, Peter Esser, Michael Friedrich, Jochen Ernst, Uwe Platzbecker, Vladan Vucinic, Anja Mehnert-Theuerkauf

**Affiliations:** ^1^Department of Medical Psychology and Medical Sociology, Comprehensive Cancer Center Central Germany (CCCG), University Medical Center Leipzig, Leipzig, Germany; ^2^Medical Clinic and Policlinic 1, Hematology and Cellular Therapy, Leipzig University Hospital, Leipzig, Germany

**Keywords:** cancer, compliance, hematological cancer, posttraumatic stress disorder, adjustment disorder

## Abstract

**Background:**

Hematological cancer patients must comply with extensive medical instructions to prevent cancer progression or relapse. Psychological comorbidities and patient characteristics have been shown to affect compliance. However, the impact of posttraumatic stress disorder (PTSD) and adjustment disorder (AjD) on compliance in cancer patients remains unclear. This study aims to evaluate compliance in hematological cancer patients more comprehensively and to investigate its association with PTSD and AjD symptomatology as well as sociodemographic and medical factors.

**Methods:**

Hematological cancer patients were cross-sectionally assessed via validated questionnaires for PTSD (PCL-5) and AjD (ADMN-20), and three internally developed items on compliance with medical regimen, with two referring to compliance behavior and one item assessing perceived difficulties with complying. Each compliance item was analyzed descriptively. Multiple linear regression models tested the association between compliance and PTSD and AjD symptomatology, sociodemographic and medical factors.

**Results:**

In total, 291 patients were included (response rate 58%). Nine out of ten patients reported to either never (67%) or rarely (25%) change their medical regimen. However, 8% reported to change it once in a while or often. Compliance behavior was mostly rated as very easy (36%) or easy (45%) to implement. Nevertheless, 19% perceived it to be partly difficult or difficult to follow medical regimen. Symptoms of AjD (β = 0.31, *p* < 0.001) were associated with more difficulties to comply. Higher compliance behavior in turn was associated with stem cell transplantation (SCT) treatment (β = −0.21, *p* < 0.001) and lower education (β = −0.19, *p* = 0.002).

**Conclusion:**

Although most patients indicated that they comply with medical regimen, a considerable subgroup of patients indicated subjectively perceived difficulties and thus seem to require additional support in implementing medical instructions possibly through improved medical communication and patient health literacy or shared decision-making.

## Introduction

Hematological cancer patients are often treated with permanent or self-administered medication (Weingart et al., 2008) and must follow medical regimen to prevent severe complications, disease progression or relapse. Compared to other cancer types, medical regimen for hematological cancer patients often contains more complex or aggressive treatments, such as a stem cell transplantation (SCT), in addition to complex schedules of multiple drugs over a long period of time, spatial isolation and hygienic standards after receiving a SCT (Sullivan et al., 2001). Non-compliance with medical treatment has been found to increase healthcare utilization, hospitalization and medical costs (Wu et al., 2009), in addition to affecting the outcome and mortality of the underlying cancer disease (Simpson et al., 2006; Pinquart and Duberstein, 2010; Zhu et al., 2017), especially in patients after SCT (Prieto et al., 2005; Harashima et al., 2019). Compliance with medical regimen, also called *adherence* in the literature and to be used synonymously here (DiMatteo et al., 2000), is therefore of particular interest in hematological cancer patients. Previous studies and systematic reviews focusing on hematological cancer patients report compliance rates between 20 and 98% (Hall et al., 2016; Bouwman et al., 2017), depending on the definition of compliance, e.g., mean compliance scores, categories, or percentages of fully compliant patients. Several sociodemographic and medical factors have been found to be associated with compliance, like age, sex, education, treatment-related side effects or physical comorbidities (Verbrugghe et al., 2013; Puts et al., 2014). However, previous studies on compliance mainly focused on medication compliance, which does not encompass extensive medical regimen faced by hematological cancer patients, e.g., following hygienic standards at home after SCT. Compliance thus needs to be approached as a broader construct.

Due to severely stressful events faced by many cancer patients during cancer diagnosis and intensive treatment, high levels of distress and symptoms of posttraumatic stress disorder (PTSD) or adjustment disorder (AjD) are prevalent comorbidities (Mehnert and Koch, 2007; Smith et al., 2008; Mitchell et al., 2011; Abbey et al., 2015; Van Beek et al., 2022). Several sociodemographic and medical factors have been shown to influence frequency and severity of PTSD and AjD symptomatology. Most commonly reported factors comprise more invasive treatments, physical comorbidities, younger age, advanced types of cancer and lack of social support (Smith et al., 2008; Cordova et al., 2017; Jung et al., 2021). We recently demonstrated with the same sample of hematological cancer patients that one in three patients suffers from clinically relevant levels of cancer-related PTSD or subthreshold PTSD, and more than half met at least one core symptom cluster of AjD (Springer et al., 2023). Symptoms related to PTSD or AjD, such as avoidance of cancer-related stressors, might influence patients’ health behavior, e.g., compliance with self-administered medication. Psychological comorbidities in cancer patients have been shown to reduce compliance with cancer treatment (DiMatteo et al., 2000; Theofilou and Panagiotaki, 2012; Arrieta et al., 2013; Bouwman et al., 2017; Haskins et al., 2019). This association is often adduced as a factor moderating the effect of psychological comorbidities on health-related outcomes or cancer mortality (DiMatteo et al., 2000; Pinquart and Duberstein, 2010). Previous studies in cancer patients, however, mainly focused on depression and anxiety (DiMatteo et al., 2000; Theofilou and Panagiotaki, 2012; Arrieta et al., 2013; Bouwman et al., 2017; Haskins et al., 2019). Even though studies on populations other than cancer patients, e.g., stroke survivors (Kronish et al., 2012) or patients with human immunodeficiency virus (HIV) (Delahanty et al., 2004), have investigated PTSD and compliance, comparable data on cancer populations are lacking to date. Nevertheless, the importance of PTSD and AjD treatment in cancer patients is often attributed to its impact on compliance.

Therefore, the aims of the present study were (i) to evaluate rates of compliance with medical regimen in hematological cancer patients and (ii) to investigate the association between compliance and PTSD and AjD symptoms, as well as with sociodemographic and medical factors.

## Methods

### Study design and participants

Hematological cancer patients were included in this cross-sectional study from different wards and the outpatient unit of the Clinic for Hematology, Cellular Therapy and Hemostaseology at the University Medical Center Leipzig. Inclusion criteria were (i) diagnosis of any hematological malignancy, myelodysplastic syndrome, or other neoplasms of the lymphoid, hematopoietic and related tissues (ICD-10: C81-C96, D46-47), (ii) aged 18−70, (iii) no planned re-admission at the time of study inclusion, (iv) fluent in German language, and (v) cognitively able to provide informed consent. Patients with SCT were only included if their most recent SCT was less than 2 years before study inclusion in order to capture relevant treatment-related stressors. All included patients provided written informed consent. The study protocol was approved by the ethics committee of the Medical Faculty at the University of Leipzig (447/17-ek).

Recruitment took place from April 2019 to September 2021. In a first recruitment wave, attending physicians approached eligible patients at inpatient wards and asked for their agreement to be contacted by a member of the study team. Additionally, patients were recruited after the study was presented at a patient congress and advertised in the network of two German blood cancer organizations. During the study phase, the recruitment procedure was adapted due to the COVID-19 pandemic in order to reduce personal contacts and the workload of physicians. Subsequently in the second recruitment wave, a study member screened all patients scheduled for the outpatient clinic for their eligibility via the clinics documentation system. All eligible patients were then contacted by the study team via phone or mail, were informed about the study and asked for their participation. The sample is thus based on convenience sampling, and non-responder analyses were computed to indicate a possible bias of the sample. Patients who declined study participation were asked about their reason for non-participation.

### Data collection

Participants were assessed via paper-pencil questionnaire at home and a structured clinical interview over the phone or in person. If the questionnaire was missing, participants were reminded regularly by phone for up to 5 times. The assessment was done at a minimum of 6 weeks after the end of treatment to ensure that there were no more acute treatment-related stressors. Patients receiving a permanent treatment were assessed directly after study inclusion. An incentive of 20 Euros was paid to participants who completed the study assessment.

### Outcomes

Compliance with medical regimen was assessed via self-report with three internally-developed items used before by our research group (Schröder and Ernst, 2008), since we did not find an adequate questionnaire for this purpose that more comprehensively addressed compliance with all kinds of medical regimen directed at hematological cancer patients. Compliance behavior was assessed with two items, one for *general compliance* behavior (“Overall, how do you rate your compliance with medical regimen?”), rated on a visual analog scale (0 = I do not comply with medical regimen at all, 100 = I fully comply with medical regimen), and the second for *modifications of medical regimen* on the patients’ own initiative (“Have you ever independently changed your medical regimen, e.g., the dose of medication or the period of taking it?”) with answers on a 5-point Likert scale (0 = never, 1 = rarely, 2 = once in a while, 3 = often, 4 = all the time). The second item was inversely recoded, so that higher values reflect higher compliance. The third item assessed subjectively *perceived difficulties with complying* with medical regimen (“Compliance with important medical regimen for me is usually…”), rated on a 5-point Likert scale (0 = very easy, 1 = easy, 2 = partly easy partly difficult, 3 = difficult, 4 = very difficult), with higher scores indicating greater perceived difficulties.

Posttraumatic stress disorder (PTSD) symptomatology was assessed with the Posttraumatic Stress Disorder Checklist (PCL-5) (Blevins et al., 2015; Krüger-Gottschalk et al., 2017). Twenty items are rated on a 5-point Likert scale (0 = not at all, 4 = extremely), with a sum score ranging from 0 to 80. Higher values indicate higher symptomatology. The scale shows good reliability and validity (Blevins et al., 2015; Krüger-Gottschalk et al., 2017). PTSD symptoms were assessed only in relation to cancer-related stressful events.

Adjustment disorder symptomatology was assessed with the 20 item Adjustment Disorder New Module (ADNM-20) (Glaesmer et al., 2015; Lorenz et al., 2016), whereby 19 items assess AjD symptoms, and one item refers to the functional impairment caused by the symptoms. All items are rated on a 4-point Likert scale (1 = never, 4 = often), with a sum score ranging from 20 and 80. Higher values indicate higher symptomatology. The scale shows good reliability and validity (Glaesmer et al., 2015; Lorenz et al., 2016). AjD symptoms were assessed only in relation to cancer-related stressful events.

Data on sociodemographic and medical variables were extracted from medical charts (i.e., age, sex, diagnosis, date of diagnosis, relapse, and SCT treatment) and via self-report from the questionnaire (i.e., education, employment, partner, remission, and cancer treatment).

### Statistical analysis

The sample characteristics were displayed descriptively. Non-responder-analyses via Chi-square- and independent *t*-test could be performed among all patients screened at the hematological outpatient clinic.

Compliance with medical regimen, i.e., *general compliance*, *modifications of medical regimen* and *perceived difficulties with complying*, were described descriptively for the total sample. Spearman correlation coefficients between all compliance items were reported with small, medium and large correlations at ≥0.10, ≥0.30, and ≥0.50, respectively, (Cohen, 1992).

To investigate associated factors with compliance, a multistep approach was applied. First, we applied separate linear regression models to identify relevant factors associated with compliance. For this, we investigate the association between the three compliance items as dependent variables and PTSD and AjD symptoms, as well as medical and sociodemographic factors as independent variables, i.e., PTSD (sum score), AjD (sum score), age (in years), sex (male vs. female), education (≤10 years vs. >10 years), remission (remission vs. active disease), relapse (yes vs. no), SCT treatment (yes vs. no), and number of physical comorbidities. Then, significant factors (*p* < 0.05) from univariate regression models were entered into multiple linear regression models for each compliance item separately, to test the importance of associated factors controlled for the others. Effect sizes for independent variables were reported as Cohen’s f^2^ (small ≥ 0.02, medium ≥ 0.15, large ≥ 0.35) (Cohen, 1992).

All statistical tests were two-tailed, with a significance level of 5%. Analyses were conducted using R (version 4.1.0) (R Core Team, 2021).

## Results

### Sample characteristics

In total, 291 hematological cancer patients were included in the study [response rate 58%; for more details on the participant flow, see previous publication (Springer et al., 2023)]. In total, 174 (60%) were male and the average age was 55 years (Table 1). Responders and non-responders did not differ in relevant sociodemographic and medical variables, i.e., age, sex, diagnosis, time since diagnosis, SCT treatment. Thirty-two patients (12%) had a relapse diagnosis and the average time since diagnosis was 2 years. A total of 285 patients provided questionnaire data and were considered for further analyses.

**TABLE 1:** Sample characteristics.

	Total sample (*n* = 291)
Age in years; mean (SD)	54.5 (12.5)
**Gender**	
Female	117 (40.2)
Male	174 (59.8)
**Education**	
≤10 years	131 (48.3)
>10 years	140 (51.7)
**Employment**	
Currently working	119 (40.9)
Not working because of cancer	83 (28.5)
Retired	69 (23.7)
Unemployed	12 (4.1)
**Partner**	
Yes	204 (79.4)
No	53 (20.6)
**Diagnosis**	
Hodgkin lymphoma	19 (6.5)
Non-follicular lymphoma	38 (13.1)
Multiple myeloma	67 (23.0)
Lymphoid leukemia (acute and chronic)	29 (10.0)
Myeloid leukemia (acute and chronic)	67 (23.0)
Myelodysplastic syndrome	26 (8.9)
Other	45 (15.5)
Time since diagnosis in months; mean (SD), median	24.3 (33.9), 13
Remitted	104 (40.6)
Relapse	32 (11.7)
Days in hospital; mean (SD), median	70.5 (67.3), 55
Days in isolation; mean (SD), median	27.0 (16.5), 25
**Treatment**	
Chemotherapy	231 (79.4)
Radiation	56 (19.2)
Antibody	56 (19.2)
Surgery	33 (11.3)
SCT (allogeneic and/or autologous)	161 (55.3)
Other treatment	47 (16.2)

This table has been published in a similar form elsewhere (Springer et al., 2023); Displayed *n* and %, if not otherwise noted; most variables were self-reported in the questionnaire, thus percentages are based on valid answers in the questionnaire and sometimes not the full sample; SCT, stem cell transplantation; SD, standard deviation.

### Compliance with medical regimen

General compliance behavior showed a median value of 97 with a range from 16 to 100. Altogether, 94% of the patients scored ≥80. Regarding modifications of medical regimen, nine out of ten patients reported to either “never” (67%) or “rarely” (25%) change any medical regimen on their own initiative (Table 2). The remaining patients reported to change medical regimen “once in a while” or “often.” Complying with medical regimen was rated by four out of five patients as either “very easy” (36%) or “easy” (45%). However, one out of five patients indicated to find it at least “partly difficult” to comply with medical regimen. General compliance and modifications of medical regimen correlated (Spearman correlation) with *r* = 0.49 (*p* < 0.001), whereas perceived difficulties with complying negatively correlated with general compliance (*r* = −0.27, *p* < 0.001) and with modifications of medical regimen (*r* = −0.22, *p* < 0.001).

**TABLE 2:** Compliance with medical regimen – item scores.

Modifications of medical regimen		Perceived difficulties with compliance	
Never	183 (67)	Very easy	98 (36)
Rarely	68 (25)	Easy	121 (45)
Once in a while	17 (6)	Partly easy partly difficult	43 (16)
Often	5 (2)	Difficult	7 (3)
All the time	0 (0)	Very difficult	1 (0)

Displayed *n* (%).

### Stressor-related symptoms, sociodemographic and medical factors associated with compliance

In the univariate regression model, general compliance behavior was predicted by older age and SCT treatment. SCT remained significant in the multiple regression model with small effect size (Table 3). Less frequent modifications of medical regimen were predicted in the univariate regression model by older age, SCT treatment and lower education, whereby SCT and education remained significant in the multiple regression model when controlled for other factors, with small effect sizes.

**TABLE 3:** Association between stressor-related symptoms, sociodemographic, medical factors, and compliance with medical regimen.

	Univariate regression				Multiple regression			
	**β**	**SE**	**f^2^**	***p***	**β**	**SE**	**f^2^**	***p***
**General compliance behavior**								
Age	0.14	0.05	0.02	**0.02**	0.11	0.05	0.01	0.07
SCT (yes vs. no)	−0.18	0.66	0.03	**0.003**	−0.15	0.68	0.02	**0.01**
**Modifications of medical regimen** ^a^								
Age	0.17	0.00	0.03	**0.006**	0.09	0.00	0.01	0.14
SCT (yes vs. no)	−0.22	0.04	0.05	**<0.001**	−0.21	0.04	0.04	**<0.001**
Education (≤10 years vs. >10 years)	−0.23	0.08	0.06	**<0.001**	−0.19	0.08	0.04	**0.002**
**Perceived difficulties with compliance**								
PTSD sum score	0.31	0.00	0.10	**<0.001**	0.01	0.01	0.00	0.94
AjD sum score	0.36	0.00	0.15	**<0.001**	0.31	0.01	0.04	**0.001**
Sex (female vs. male)	−0.13	0.10	0.02	**0.03**	−0.07	0.10	0.01	0.21
Physical comorbidities	0.22	0.01	0.05	**<0.001**	0.08	0.01	0.01	0.21

Displayed all significant factors from univariate regression models that were then entered into multiple regression models; significant values (*p* < 0.05) marked in bold; ^a^higher values reflect less modifications of medical regimen; SCT, stem cell transplantation; PTSD, posttraumatic stress disorder; AjD, adjustment disorder; β, standardized regression coefficient; SE, standard error; f^2^, Cohens’ f^2^.

Greater perceived difficulties with complying were predicted in the univariate regression models by elevated symptoms of PTSD and AjD, as well as female sex and physical comorbidities. AjD symptomatology remained significant in the multiple regression model when controlled for the other factors, with medium effect size.

## Discussion

This cross-sectional study investigated compliance with medical regimen, its association with PTSD and AjD symptomatology, as well as with sociodemographic and medical factors in a sample of hematological cancer patients. The vast majority of the patients reported high overall compliance behavior, and only few mentioned having independently changed their medical regimen. However, one fifth of the patients reported at least some difficulties with complying with the medical regimen they had received. In addition, patients with elevated symptoms of AjD and PTSD reported greater perceived difficulties with compliance, even though their actual compliance behavior had not been affected. Furthermore, patients who had received a SCT tended to be more compliant than patients without SCT.

Studies among hematological cancer patients have reported that about 50% of the participants had been fully compliant with their medication (Hall et al., 2016; Bouwman et al., 2017). This corresponds well with our results, which show that half of the patients scored 97 or higher on a scale from 0 to 100 for general compliance. Nevertheless, our results revealed no association between symptoms of PTSD or AjD with compliance behavior. A meta-analysis had previously linked medication compliance behavior with other psychological comorbidities, with mixed results showing that cancer patients suffering from depression were more likely to be non-compliant; however, such an association was not detected with anxiety (DiMatteo et al., 2000). A recent study among 472 hematological cancer patients found no effect of depression and anxiety on medication compliance (Bouwman et al., 2017). This corresponds well with our results indicating no association between PTSD and AjD symptomatology and compliance behavior. Even though many studies outline the necessity to treat stressor-related symptoms in cancer patients due to their perceived impact on compliance, empirical studies on PTSD and AjD were lacking. We thus aimed to expand existing evidence on the link between psychological comorbidities and compliance in cancer patients. Although we did not observe an association between compliance behavior and PTSD or AjD, this does not necessarily imply that there is no effect on compliance. For instance, different coping mechanisms as a response to trauma and severely stressful situations may cancel each other out. On the one hand, there may be avoidance of the stressor, leading to reduced compliance behavior, or, on the other hand, the response may be approaching the stressor and increasing one’s focus, leading to highly compliant behavior (Roth and Cohen, 1986). Future research investigating trauma coping mechanisms in relation to compliance behavior is necessary to test this hypothesis.

Difficulties with complying with medical regimen had not been investigated in previous studies and, in our results, showed only small correlations with compliance behavior. One explanation for our result of increased perceived difficulties in patients with cancer-related AjD and PTSD might be that cancer patients are constantly reminded of their cancer-related stressors by internal bodily signs and situations in which they need to follow their medical regimen. This may lead to a persistent increase of psychological distress with little possibilities to avoid stressors, which might affect the subjectively perceived difficulty to act upon medical cancer-related regimen. A similar effect might occur in patients with additional physical comorbidities, which have been shown to increase difficulties with compliance in our results when tested independently. However, physical comorbidities and PTSD did not remain significant when adjusting for other medical and psychological factors and results should thus be interpreted with caution. The different picture in associated factors for compliance behavior and perceived difficulties to comply underlines the necessity for the development of specific assessment tools for compliance in hematological cancer patients that capture compliance and relevant barriers in more detail.

Although there is extensive research investigating compliance rates and determinants in hematological cancer patients after SCT (Morrison et al., 2017), a systematic comparison of patients with and without SCT is lacking. Our results indicate higher compliance in patients with SCT. This might be explained by their medical history of cancer treatment, including longer time spent in hospital and an urgent need to follow medical regimen to avoid life-threatening complications. It may be hypothesized that medical regimen for SCT recipients is introduced by treating physicians in more detail and with greater importance attached to them, compared to hematological cancer patients receiving other cancer treatments. Contrary to our results showing less educated people to be more compliant, a study among 169 patients with chronic myeloid leukemia found higher education levels to be associated with better medication compliance (Noens et al., 2009). However, they assessed general compliance, regarding which we did not observe an effect of education. Modifications of medical regimen, that were associated with level of education in our study, may represent a different part compliance behavior. One explanation for our results may be that patients with lower education are less likely to modify their medical regimen, as this would represent an action and decision contrary to the physician’s medical opinion. Such independent decision-making requires sufficient health literacy, which has been shown to be reduced in patients with lower education (Van Der Heide et al., 2013).

### Clinical implications

Even though most patients reported compliance with medical regimen and did not change the prescribed medical regimen, there is a substantial part of patients indicating at least some difficulties with compliance. It may therefore be valuable for physicians to address potential difficulties already at the point of introducing medical regimen. In this context, it may be useful to identify the nature of these difficulties, e.g., reluctance due to side effects of the medication, forgetting or repressing medical regimen to avoid being constantly reminded of the disease. This knowledge may help to overcome barriers and provide individualized support to patients in need. In addition to the burden caused by symptoms of PTSD and AjD, their identification and treatment in cancer patients seems to be crucial given perceived difficulties in compliance, as medical regimen may be perceived as an additional stressor by an already distressed patient. In terms of actual compliance behavior, patients with SCT seem to be more aware of the necessity to comply and are more compliant as a result. Additional attention should be given to patients without SCT to improve compliance behavior in this population.

### Strengths and limitations

To our knowledge, this study is the first to investigate the relation between compliance with PTSD and AjD symptomatology in hematological cancer patients. Valid medical data were available from the medical records. Furthermore, compliance was assessed more comprehensively than in previous studies that mostly focused on medication compliance. Thus, we provide a broader insight into compliance with medical regimen specific to patients with hematological malignancies.

There are several limitations to be mentioned. First, compliance was assessed via internally developed single-items due to a lack of appropriate assessment tools. Future research needs to develop and evaluate standardized assessment tools of comprehensive compliance. Second, since hematological cancer patients face extensive medical regimen, which do not necessarily apply to other cancer entities, and show specific cancer-stressors that may lead to PTSD and AjD, generalizability of our results to other cancer populations is compromised. However, unique demands, stressors and medical regimen for hematological cancer patients require an evaluation of specifically this cancer population. Third, the cross-sectional design does not allow any causal conclusions regarding the association of compliance with stressor-related symptoms and patient characteristics. Since this was an explorative study, further research using longitudinal data is, however, needed to confirm or disprove our results.

### Conclusion

Compliance with medical regimen in hematological cancer patients is high in the vast majority of the patients, especially in SCT recipients. However, subjectively perceived difficulties with complying with medical regimen are not uncommon, as reported by one fifth of the study participants. Specific assessment tools for comprehensive compliance are needed to better understand potential barriers and support patients in need with their compliance. Symptoms of AjD and PTSD might exacerbate distress in situations where patients are required to comply to a medical instruction. However, overall compliance behavior remained unaffected within this hemato-oncological sample with a complex but critically important medical regimen to follow. The subgroup of patients with difficulties with compliance and those at risk of non-compliance may require further support with medical regimen, possibly by improving medical communication and patient health literacy or prompting shared decision-making.

## Data availability statement

The raw data supporting the conclusions of this article will be made available by the authors, without undue reservation.

## Ethics statement

The studies involving humans were approved by the Ethics committee Medical Faculty University of Leipzig. The studies were conducted in accordance with the local legislation and institutional requirements. The participants provided their written informed consent to participate in this study.

## Author contributions

FS: Data curation, Formal analysis, Investigation, Methodology, Project administration, Writing – original draft, Writing – review and editing, Conceptualization. PE: Conceptualization, Data curation, Funding acquisition, Investigation, Methodology, Writing – review and editing. MF: Data curation, Methodology, Writing – review and editing. JE: Conceptualization, Investigation, Methodology, Writing – review and editing. UP: Resources, Writing – review and editing. VV: Resources, Writing – review and editing. AM-T: Conceptualization, Funding acquisition, Methodology, Supervision, Writing – review and editing.
